# Coding-complete genome sequence of cucumber green mottle mosaic virus in Iraq

**DOI:** 10.1128/mra.00354-26

**Published:** 2026-06-22

**Authors:** Athraa A. Hadi, Mushtak T. Mohammadali, Sienaa M. Al-Zurfi, Adnan A. Lahuf

**Affiliations:** 1 Plant Protection Department, Agriculture College, University of Kerbala 125663, Karbala, Iraq; Katholieke Universiteit Leuven, Leuven, Belgium

**Keywords:** cucumber green mottle mosaic virus, complete coding genome, Iraq

## Abstract

This study presents the coding-complete genome sequence of cucumber green mottle mosaic virus from Iraq. The complete genome is 6,398 nucleotides, containing four open reading frames, with a 5′ untranslated region (UTR) of 46 nt and a 3′ UTR of 163 nt.

## ANNOUNCEMENT

The cucumber green mottle mosaic virus (CGMMV), which is currently named *Tobamovirus viridimaculae*, belongs to the genus *Tobamovirus* in the family *Virgaviridae* (1). It possesses a single-stranded, positive-sense RNA genome (1, 2). Its primary hosts are crop species within the *Cucurbitaceae* family, where it induces symptoms such as leaf mottling and mosaic on foliage, plant stunting, flesh discoloration, surface mottling, and fruit distortion (3). Severe reductions in fruit yield and quality can occur in susceptible cucurbit crops, including watermelon and cucumber (4). The virus was first reported in Europe (5–7), Asia (8–10), and the Middle East (11) and has since reached North America (12, 13).

In 2025, symptomatic leaf samples with signs of thrips infestation were collected from cucumber plants exhibiting leaf mottle and distortion symptoms in commercial plastic houses in northern and central Iraq. A subset of these symptomatic leaf samples, along with the associated thrips insects, was pooled and subjected to metatranscriptomic sequencing at BGI (Hong Kong, China). Based on the company’s protocol, total RNA was extracted and treated with DNase I to remove DNA contaminants. Ribosomal RNA was depleted using the Ribo-off rRNA Depletion Kit, followed by RNA fragmentation. First-strand and second-strand cDNA synthesis was performed using random hexamer primers, with dUTP replacing dTTP during second-strand synthesis. The cDNA fragments were end-repaired, adenylated, adaptor-ligated, and PCR-amplified. After library quality control, the PCR products were denatured, cyclized, converted into DNA nanoballs (DNBs), and sequenced on the DNBSEQ platform.

A total of 36,126,439 raw reads (100 nt length) were obtained. Trimmomatic software (v.0.32) was applied for trimming the raw paired-end reads. A total of 126,534 reads that did not map to the reference genome sequence of *Cucumis sativus* (cucumber) (GCF_000004075.3) using Bowtie2 software (v.2.5.5) with default parameters were *de novo* assembled using Velvet assembler (v.1.2.10) following their default parameters (14). The resulting contigs were analyzed using BLASTn against documented plant virus sequences retrieved from GenBank (https://www.ncbi.nlm.nih.gov/genome/viruses/) (15).

This analysis revealed >99% pairwise nucleotide identity and 100% coverage with various global isolates of CGMMV. Thus, the reference sequence of CGMMV (NC_001801) was used for reference-based assembly using Geneious Prime 2024.0.4 (Biomatters) for validation and annotation. The complete genome of the CGMMV isolate obtained was 6,398 nucleotides (nt) in length, containing four open reading frames with a 5′ untranslated region (UTR) of 46 nt and a 3′ UTR of 163 nt with GC content 42.4% and 100% coverage. BLAST analysis (16) showed that the closest genomic nucleotide identities were 99.77% to isolate MH271420.1 from the Netherlands, 99.70% to PX021799.1 from China, and 99.70% to MH271408.1 from the USA.

To confirm the presence of CGMMV, a specific RT-PCR assay was successfully employed using primers flanking the capsid and movement protein genes: forward primer (5′-CATAGATGTCTCTAAGTAAGGTG-3′; position 4974 bp) and reverse primer (5′-CCCTCGAAACTAAGCTTTCG-3′; position 6243 bp) (17). This assay was applied to the remaining symptomatic leaf samples as well as newly collected samples from the same commercial cucumber plastic houses. It was found that 45% of the remaining leaf samples were infected with this virus.

Several viruses infecting cucurbit crops have been reported in Iraq (18, 19). However, to the best of our knowledge, this study represents the complete genome sequence of CGMMV in Iraq. This information will be crucial for developing and implementing effective disease management strategies.

## Data Availability

The coding-complete genome sequence of CGMMV-Iraq1 has been deposited in GenBank under accession number PZ167520.1. The BioProject accession number is PRJNA1440257. The BioSample accession number for this isolate is SAMN56608350. The raw sequence data related to the virus were deposited in the NCBI Sequence Read Archive (SRA) under accession number SRR37706242. All records can be accessed through the NCBI database at https://www.ncbi.nlm.nih.gov.
